# Consumption and Malaria

**Published:** 1853-09

**Authors:** G. R. Grant

**Affiliations:** Memphis, Tenn.


*Consumption and Malaria.* By G. R. Grant, M.D., Memphis, Tenn. It is believed, and has been asserted by high authority, that *phthisis pulmonalis* is not only not so prevalent in malarious as in non-malarious regions, but, also, that by a residence in localities confessedly abounding with malaria, the consumptive invalid migkt reasonably expect a permanent cure. An inspection of the foregoing table will show, as far as it goes, that facts are not in accordance with this opinion. *Pulmonary consumption*, as well as *typhoid fever*, is undeniably on the increase in Memphis, where *malaria* is almost as abundant as around the "Pontine Mountains."

Nor is this all. An examination of the preceding table likewise informs us that *consumption* has not only consigned victims to our cemeteries during every month of the past year, but that exactly *one-half* of the whole number of deaths caused by it occurred from July to October inclusive, the very season of the year when *malaria* is in most abundance, and is exerting its power to the fullest extent.

No one will run the risk of making himself the subject of ridicule, by asserting that these cases are *strangers* who have come hither to spend their *summers* on account of the healthfulness of the place.

As far as the facts furnished by one malarious locality can be depended on, it may be asserted that an exemption from pulmonary consumption is not attainable by a residence in a sickly place, much less ought a cure to be expected under such circumstances.—*American Journal of the Medical Sciences.*

